# Mangiferin Inhibits Human Lung Adenocarcinoma by Suppressing MiR-27b and MiR-92a

**DOI:** 10.1155/2021/2822950

**Published:** 2021-07-12

**Authors:** Xiao-Jv Chi, Jian-Jun Meng, Chun-Yu Lin, Qi-Sheng Su, Yuan-Yuan Qin, Ru-Hua Wei, Dong Lan, Chao Huang

**Affiliations:** ^1^Department of Clinical Laboratory, First Affiliated Hospital of Guangxi Medical University, 6 Shuangyong Road, Nanning, Guangxi Zhuang Autonomous Region 530021, China; ^2^Department of Geriatrics, First Affiliated Hospital of Guangxi Medical University, 6 Shuangyong Road, Nanning, Guangxi Zhuang Autonomous Region 530021, China; ^3^Department of Radiation Oncology, First Affiliated Hospital of Guangxi Medical University, 6 Shuangyong Road, Nanning, Guangxi Zhuang Autonomous Region 530021, China; ^4^Department of Medical Oncology, First Affiliated Hospital of Guangxi Medical University, 6 Shuangyong Road, Nanning, Guangxi Zhuang Autonomous Region 530021, China; ^5^School of Information and Management, Guangxi Medical University, 22 Shuangyong Road, Nanning, Guangxi Zhuang Autonomous Region 530021, China

## Abstract

Lung adenocarcinoma (LUAD) is one of the most prevalent malignancies. However, its mechanism and therapeutic strategy remain to be clarified. Mangiferin is a flavonoid derived from the leaves of mango trees of the lacquer family that has many pharmacological and physiological effects. This research aimed to elucidate the biological effect of mangiferin in LUAD cell lines and clarify the in vitro mechanism of mangiferin. Mangiferin was shown to significantly restrain the proliferation of LUAD cells (A549, H1299, and H2030 cells) in a dose- and time-dependent manner. Furthermore, mangiferin was capable of stimulating apoptosis, and more cells were blocked in G1 and S phase in the mangiferin-treated cells than in those not treated with mangiferin. Microarrays and micro-RNA sequencing data suggested that there is a higher level of miR-27b and miR-92a in LUAD tissues than in non-LUAD tissues. Additional experiments indicated that mangiferin may be related to the downregulated levels of miR-92a and miR-27b. In conclusion, mangiferin likely regulates proliferation and apoptosis in LUAD cells by reducing the expression levels of miR-92a and miR-27b.

## 1. Introduction

Lung cancer (LC), a highly heterogeneous malignancy, contributes to nearly a quarter of cancer-related deaths globally, with almost 45% of cases being lung adenocarcinoma (LUAD) [1, 2]. Over 60% of LC patients are diagnosed at a locally metastatic or advanced stage owing to the lack of techniques and recognizable symptoms for early detection, and conventional surgery may not be a useful option for these patients. Although great progress in therapeutic strategies has improved the prognosis for some LUAD patients, the overall survival within 5 years is still less than 20% [3]. Thus, further investigation into novel agents for LUAD and their mechanisms is urgently needed.

Polyphenols are a kind of chemical substances abundant in plants, which play important roles in plant growth and metabolism. More and more evidence show that polyphenols have potential health properties on human body, such as antioxidant, antiallergic, and anti-inflammatory [4]. To our knowledge, many kinds of polyphenols can resist the occurrence and development of tumor. For example, curcumin inhibits the proliferation and migration of tumor cells and promotes apoptosis [5]. Lingonberry and bilberry have been reported to have effects on gastrointestinal cancer [6]. Looking for new anticancer polyphenols may help to improve the current therapy.

**Figure 1:**
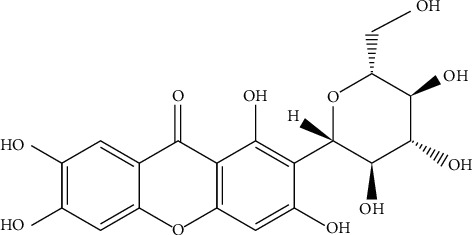
The chemical structure depiction of mangiferin (C_19_H_18_O_11_).

Mangiferin is derived from the leaves, stems, peels, and roots of *Mangifera indica* and other herbaceous plants [7] (Figure 1). Studies have described a variety of biological effects of mangiferin on the human body, including antitumor, antioxidant, antibacterial, and antiviral effects [8–11]. One in vitro experiment showed that mangiferin exerts an influence on cell cycle arrest and induces apoptosis in A549 cells [12]. In addition, several studies have suggested that mangiferin plays a reverse or inhibitory role in lung carcinogenesis [13, 14]. However, the precise molecular mechanism remains unknown.

Micro-RNAs (miRNAs) are not only important regulators of cell proliferation, differentiation, and apoptosis but also closely related to cell phenotype and human diseases [15]. Emerging evidence has indicated that upregulated miR-27b-5p (miR-27b) and miR-92a-1-3p (miR-92a) levels contribute to various types of cancers [16–19]. The overexpression of miR-92a has been shown to promote cellular activity and suppress the sensitivity of lung cancer cells to gefitinib [20–23]. MiR-27b has also been confirmed to be consistently upregulated in dysplastic tissues compared with normal tissues during gastric carcinoma development [24].

Therefore, it is hypothesized that mangiferin might perform its antitumor role by targeting miR-92a and miR-27b. In this paper, we aimed to confirm the biological function of mangiferin in LUAD cells (A549, H1299, and H2030 cells) and detect genetic alterations affecting miR-27b and miR-92a under treatment with mangiferin. Furthermore, an in silico analysis was performed with common targets of miR-27b and miR-92a to explore the underlying mechanism of mangiferin in LUAD.

## 2. Materials and Methods

### 2.1. Reagents

Mangiferin (C_19_H_18_O_11_) was obtained from the Central Laboratory of the Institute of Clinical Medicine of Guangxi Nationalities Hospital. Mangiferin is a light-yellow crystal and is slightly soluble in water, ethanol, and methanol; soluble in hot dilute methanol and ethanol; and insoluble in nonpolar solvents. It was prepared at 100 mg/mL with 2% NaHCO_3_ and packaged and frozen at 20°C. Fetal bovine serum (FBS) was purchased from HyClone (Thermo Fisher, Logan, Utah, USA).

### 2.2. Cell Lines for *In Vitro* Experiments

The H2030, H1299, and A549 (human LUAD) cell lines, obtained from the Institute of Biochemistry and Cell Biology of Chinese Academy of Sciences (Shanghai, China), were cultured in RPMI 1640 medium (Welgene, Gyeongsan, South Korea) and routinely supplemented with 10% (v/v) FBS in a 37°C incubator with 95% O_2_ and 5% CO_2_. The experimental groups were treated with different concentrations of mangiferin which dissolved in 2% NaHCO_3_. And, the control group was treated with the same amount of 2% NaHCO_3_.

### 2.3. Cell Proliferation Detection for LUAD Cell Lines

LUAD cells were seeded into 96-well culture plates, and cell proliferation was assessed by Cell Counting Kit-8 (CCK-8) assays at 12, 24, 36, 48, and 72 hours after adding 10 *μ*L of different concentrations of mangiferin (12.5, 25, 50, and 100 mg/mL). LUAD cells were incubated for 1 hour, and then a microplate reader was used to read and analyze the sample at 450 nm (OD 450). Each experiment was performed according to the manufacturer's instructions and repeated three times.

### 2.4. Cell Cycle and Apoptosis Analyses

Collected cell lines were processed with prechilled PBS and then fixed with prechilled 70% ethanol at 4°C overnight. RNase A (50 mg/mL) was added to the cell suspension at 37°C for a 30-min incubation. The cell lines were washed with PBS and further processed with 30-min staining with propidium iodide (PI, 25 mg/mL) at 37°C. The characteristics of the cell cycle were detected using a FACS Calibur flow cytometer according to the manufacturer's instructions and further analyzed by ModFit software. The comparison rate (%) was computed as the proportion of cells in the G1/S/G2 phase.

Cell apoptosis was detected by an Annexin V-FITC Apoptosis Detection Kit (BD Pharmingen, San Diego, CA). The cells were separated into four groups in each experiment: single-stained PI group, single-stained Annexin V group, PI and Annexin V double-stained group, and nonstained group. After a 15-minute incubation in Annexin V-FITC and PI, cell apoptosis was observed using a FACS Calibur flow cytometer. Each experiment was repeated three times and performed according to the manufacturer's instructions.

### 2.5. Quantitative Real-Time PCR (qRT-PCR)

Human LUAD cells (A549, H1299 and H2030 cells) were treated with different concentrations of mangiferin (25, 50, and 100 *μ*M) for 48 hours and further cultured for 24 hours. Then, qRT-PCR was performed to detect miR-27b and miR-92a expression levels in the cells. Total RNA was isolated from cells with TRIzol reagent (Invitrogen, Braunschweig, Germany), and isolated RNA was further converted into cDNA with the RevertAid First Strand cDNA Synthesis Kit (Thermo Scientific). Next, the relative expression of miR-27b and miR-92a in cells was assessed by ABI 7300 HT software, and U6 was used as an endogenous control to normalize miRNA expression. The sequences used for analysis are as follows: (miR-27b) forward 5'-AGAGCTTAGCTGATTGGTGAAC-3' and reverse 5'-GTTCACCAATCAGCTAAGCTCT-3'; and (miR-92a) forward 5'-AGGTTGGGATCGGTTGCAATGCT-3' and reverse 5'-AGCATTGCAACCGATCCCAACCT-3'.

### 2.6. Integrated Analysis of the Functional Mechanisms of MiR-27b and MiR-92a

The expression of miR-92a and miR-27b in LUAD samples was analyzed by integrating microarray data from the Gene Expression Omnibus (GEO) and micro-RNA-seq data from The Cancer Genome Atlas (TCGA). The miRWalk 2.0 database was used to predict the genes targeted by miR-27b and miR-92a [25]. The common target genes were subjected to further enrichment analysis via the DAVID database [26], and the potential regulatory network between these genes was analyzed in the STRING database (https://string-db.org/) [27].

### 2.7. Statistical Analysis

Data were processed and analyzed in SPSS 23.0 software. The expression levels of miR-27b and miR-92a are presented as the means ± SDs, and Student's *t*-test and one-way analysis of variance (ANOVA) were applied when comparing the statistical significance between different groups. The results with *P* < 0.05 were considered statistically significant.

## 3. Results

### 3.1. Mangiferin Inhibits the Proliferation of LUAD Cells

**Figure 2:**
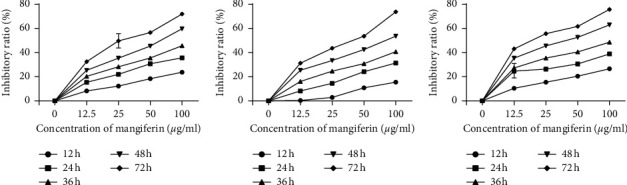
Mangiferin suppresses the proliferation of lung adenocarcinoma cells. (a) A549 cells. (b) H1299 cells. (c) H2030 cells.

To study the role of mangiferin (10, 20, and 40 *µ*M) on the proliferation of LUAD cell lines, the activity of LUAD cells was assessed by CCK-8 assay. Mangiferin showed inhibitory effects on A549, H1299, and H2030 cells, and the inhibition rate increased with increasing concentration. In addition, as the exposure time increased, the cell inhibition rate also showed an upward trend, which suggests that the inhibitory effect of mangiferin on LUAD cells is dose- and time-dependent (Figure 2).

### 3.2. Cell Cycle Distribution

**Figure 3:**
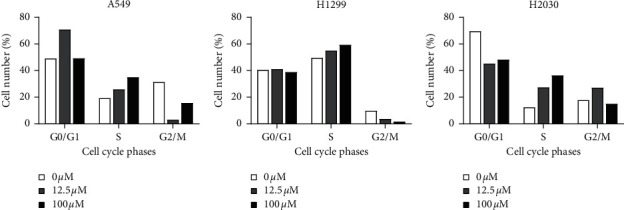
Cell cycle analysis was performed in A549 (a), H1299 (b), and H2030 (c) cells treated with mangiferin.

**Figure 4:**
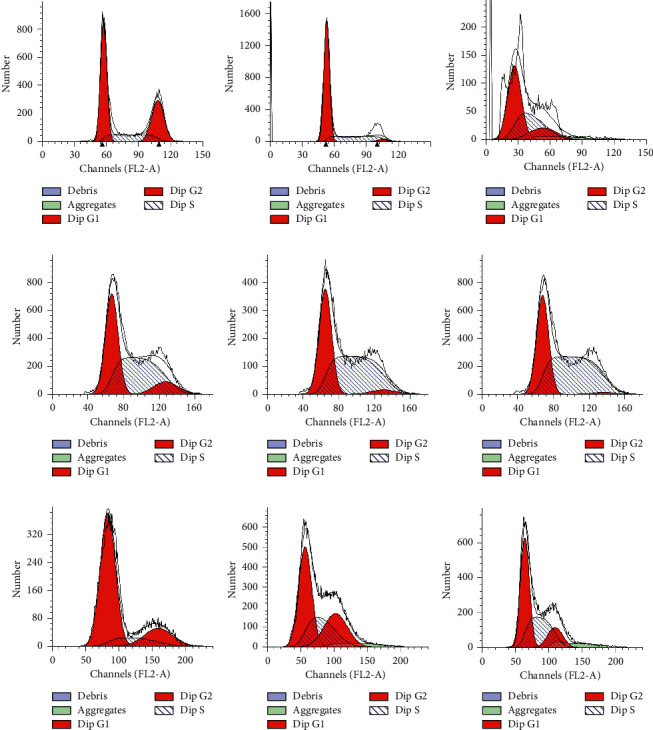
Flow cytometry was performed in A549 ((a) 0 *μ*g/ml; (b) 12.5 *μ*g/ml; (c) 100 *μ*g/ml), H1299 ((d) 0 *μ*g/ml; (e) 12.5 *μ*g/ml; (f) 100 *μ*g/ml), and H2030 ((g) 0 *μ*g/ml; (h) 12.5 *μ*g/ml; (i) 100 *μ*g/ml) cells treated with mangiferin.

After treatment with mangiferin, cell cycle analysis was performed to explore the cell cycle features of LUAD cells. Statistical analysis of G0/G1, S, and G2/M phase cell numbers showed that more cells were blocked in the G1 phase after mangiferin treatment than without mangiferin treatment, thus suggesting that mangiferin may induce A549, H1299, and H2030 cells to undergo apoptosis in a time-dependent manner (Figure 3). Moreover, S-phase arrest was discovered in the mangiferin-treated cells (Figure 4). The number of apoptotic LUAD cells (H1299, A549, and H2030 cells) after treatment with different concentrations of mangiferin (0, 12.5, and 100 mg/mL) varied with the number of apoptotic cells increasing with increasing concentration (Table 1).

### 3.3. Upregulation of MiR-27b and MiR-92a in LUAD Tissues

To explore whether miR-92a and miR-27b are differentially expressed in LUAD tissue relative to normal tissue, the GSE36681 dataset from the GEO database was downloaded for analysis [28]. The dataset contains 206 samples, of which 47 pairs of paraffin-embedded tissue and 56 pairs of fresh-frozen tissue were used. As shown in the figure, the expression levels of miR-27b and miR-92a in fresh-frozen (FF) LUAD tissues were significantly higher than those in noncancerous samples. However, consistent results were not observed in the subset of formalin-fixed, paraffin-embedded (FFPE) samples (*P* > 0.05). In addition, miRNA expression data from 495 samples (450 LUAD, 45 normal tissues) from TCGA confirmed that miR-27b and miR-92a showed higher levels in LUAD tissues than in non-LUAD adjacent tissues (Figure 4).

### 3.4. Downregulation of MiR-27b and MiR-92a after Mangiferin Treatment

**Figure 5:**
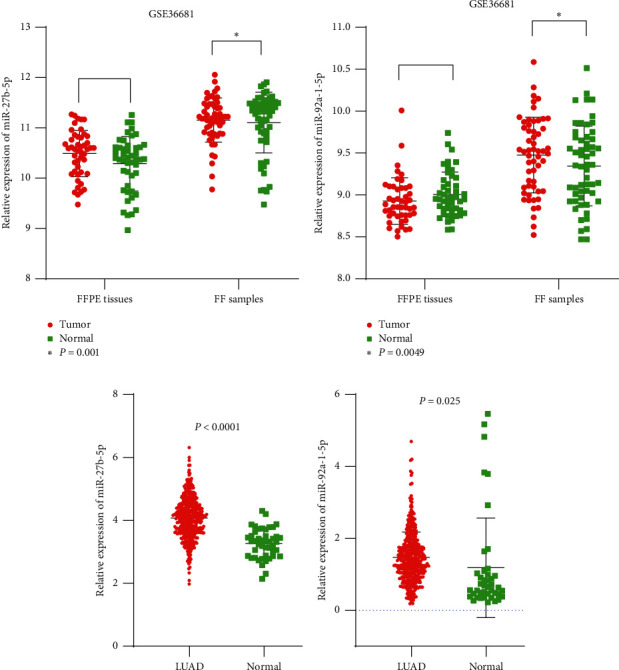
Expression of miR-27b-5p (a, c) and miR-92a-1-5p (b, d) in lung adenocarcinoma tissues from GEO and TCGA database.

**Figure 6:**
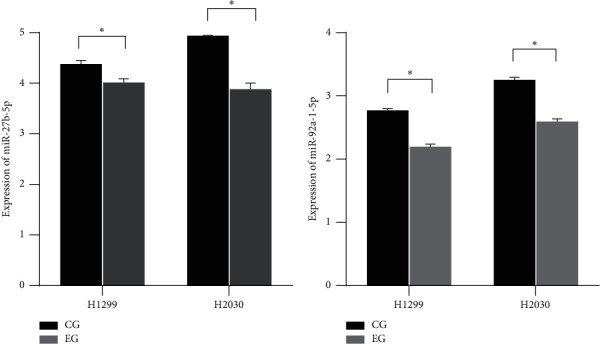
The expression levels of miR-27b-5p and miR-92a-5p in LUAD cells treated by mangiferin (CG: control group; EG: experimental group).

Analysis of TCGA and GEO LUAD expression data showed that miR-27b and miR-92a showed higher levels in LUAD samples than in normal lung tissue (Figure 5). To investigate the biological effect of mangiferin on the miR-27b and miR-92a levels, cells (H1299 and H2030 cells) were treated with mangiferin (25 *µ*M) for 24 hours, and then the levels of miR-27b and miR-92a were detected. The results suggested that miR-27b and miR-92a levels were clearly decreased in H1299 and H2030 cells after 24 hours of mangiferin treatment (Figure 6).

### 3.5. Functional Enrichment of MiR-27b and MiR-92a Target Genes and Analysis of Regulatory Networks

**Figure 7:**
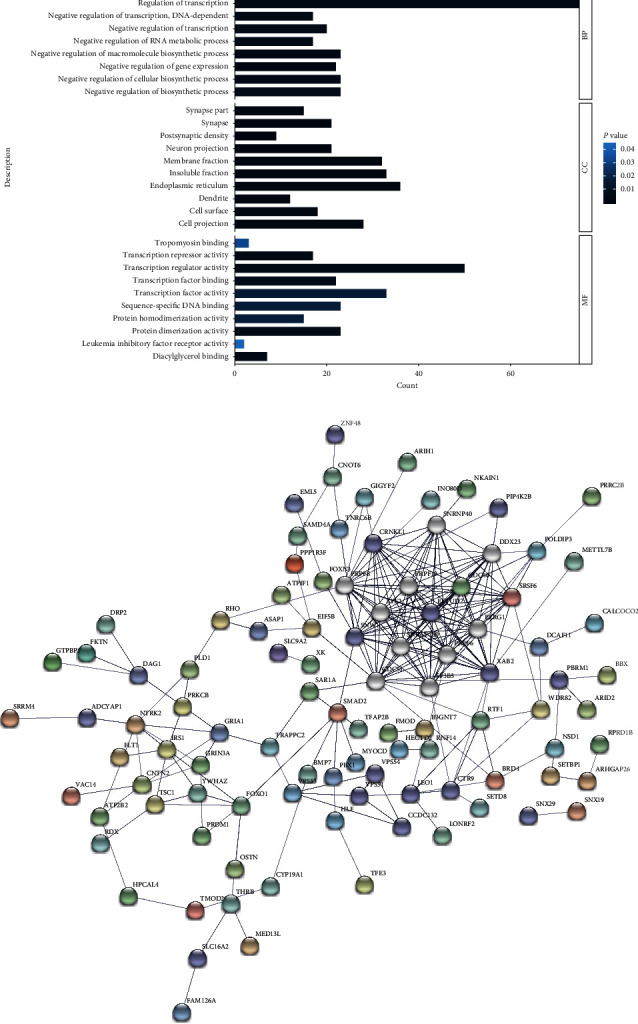
Integration analysis of potential target genes. (a) Gene Ontology analysis; (b) PPI network of genes (number of nodes: 153, number of edges: 239, PPI enrichment *P* value: <1.0*e*−16).

Potential target genes that appeared in 5 or more databases were included, from which we obtained 1196 targets of miR-27b-5p and 1589 genes targeted by miR-92a-1-5p. Finally, a total of 426 common genes were analyzed for enrichment. Gene Ontology (GO) analysis is used to determine the functions of genes at three levels: cellular component (CC), molecular function (MF), and biological process (BP) levels. The results indicated that the common target genes were mainly involved in the regulation of the RNA transcription process, and their main molecular functions included the regulation of transcriptional activity and the binding of transcription factors. Their positions in cells were mainly within the membrane fraction, insoluble fraction, and endoplasmic reticulum (Figure 7).

## 4. Discussion

This study aimed to investigate the repressive role of mangiferin and its potent mechanism in LUAD cells with the hope of identifying a novel therapeutic for LUAD patients. The present paper confirmed that mangiferin induces effects by analyzing three subsets of LUAD cells, and further exploration regarding its potent mechanism indicated that mangiferin exerts an apoptosis-inducing and growth-inhibitory effect via the suppression of miR-27a and miR-92.

Which factors are related to mortality for LUAD patients? This is a major question emerging from the rapidly increasing number of lung cancer cases. As an extremely fatal cancer, dysregulation of growth and high metastatic ability limit the probability of curing this disease. Although great achievements have been made within recent decades, the underlying mechanism of LUAD carcinogenesis is still not clear, and patients with LUAD still face a poor prognosis.

Recently, mangiferin has gained much attention for its potential ability against human cancers. Previous studies have shown that mangiferin can induce apoptosis and thus exert an antitumor effect in multiple cancerous cells, including leukemia, nasopharyngeal carcinoma, and HCC cell lines [29, 30]. Specifically, consistent results were also observed in human LUAD A549 cells, in which mangiferin has been reported to inhibit cell proliferation and induce apoptosis by inactivating cdc2-cyclin B and the NF-*κ*B signaling pathway [31]. In addition, treatment with mangiferin strengthens the ability of biological enzymes, such as diphosphate-glucuronosyl transferase, quinone reductase, and glutathione transferase, to break down toxins and lessens genome deterioration in LC-bearing animals [32].

In addition to the latent inhibitory effect of mangiferin on the growth of A549 cells, the discoveries in this paper further suggest that the administration of mangiferin leads to an important inhibition of the proliferation of three subsets of LUAD cells (A549, H1299, and H2030 cells) in a dose- and time-dependent manner. In addition, as detected by Annexin V/PI staining, our results also revealed that mangiferin is able to block the cell cycle and promote cell apoptosis in LUAD cells.

It is universally known that miRNAs exert a crucial influence on the genetic regulation network. MiRNAs have been shown to widely participate in the processes of tumorigenesis, apoptosis, and differentiation. More than half of human miRNA-related genes in the genome are located in tumor-associated regions or vulnerable sites, and in silico profiling has implied that the level of specific miRNAs might be an indicator to predict tumor differentiation status and stage. It was discovered that mangiferin possesses anticancer effects in glioma by inducing miR-15b expression, and mangiferin has been shown to inhibit proliferation by upregulating miR-182 in prostate cancer cells [10, 33]. Interestingly, the present results indicate that mangiferin treatment specifically represses the expression of miR-92a and miR-27b in LUAD cells.

A study published in 2019 showed that the upregulation of miR-92 clearly reduced the levels of the tumor suppressor gene NF2 in HCT116 and A549 cells and promoted migration, growth, and survival [34]. Additionally, another study examining a total of 2573 patients also suggested that overexpression of miR-92a serves as an unfavorable prognostic indicator in LUAD patients [35]. To our knowledge, miR-27b-5p had been reported as a tumor suppressor in ovarian carcinoma and oral cancer [20, 36], but it is highly expressed in gastric adenocarcinoma and may play a positive role in tumorigenesis and development [37]. However, regrettably, no study has reported the relationship between miR-27b-5p and lung cancer. As determined by data from TCGA and GEO microarrays, both miR-27b and miR-92a are present at a higher level in LUAD tissues than in adjacent tissues. After mangiferin treatment, a marked downregulation of miR-27b and miR-92a was observed in LUAD cells; thus, we can infer that mangiferin exerts a growth-repressive and apoptosis-inducing effect by directly targeting miR-27b and miR-92a.

However, some shortcomings of this study should be noted. First, the biological function of miR-27b and miR-92a needs to be verified in vitro. In addition, key molecules related to mangiferin-mediated apoptosis or cell cycle arrest were not detected. Moreover, clinical trials should be further performed to confirm whether mangiferin can be used to treat LUAD patients.

In conclusion, we found that mangiferin may negatively regulate the expression of miR-92a and miR-27b to influence not only cancerous growth but also the cell cycle and apoptotic capability of LUAD cells, which indicates that mangiferin is likely to be a hopeful therapeutic agent for LUAD patients. In the future, further experiments are warranted to confirm the current results.

## Figures and Tables

**Table 1:** Effects of different concentrations of mangiferin on apoptosis of lung adenocarcinoma cell lines.

Cell lines	Apoptosis rate (%)		
	0 *μ*mol/L	12.5 *μ*mol/L	100 *μ*mol/L
A549	0.56	8.99	93.13
H1299	0.06	8.97	89.93
H2030	0.14	13.31	53.23

## Data Availability

The data used in this study were obtained from open databases.
